# Identification, binding, and structural characterization of single domain anti-PD-L1 antibodies inhibitory of immune regulatory proteins PD-1 and CD80

**DOI:** 10.1016/j.jbc.2022.102769

**Published:** 2022-12-05

**Authors:** Tara Kang-Pettinger, Kayleigh Walker, Richard Brown, Richard Cowan, Helena Wright, Roberta Baravalle, Lorna C. Waters, Frederick W. Muskett, Matthew W. Bowler, Kovilen Sawmynaden, Peter J. Coombs, Mark D. Carr, Gareth Hall

**Affiliations:** 1 Leicester Institute of Structural and Chemical Biology and Department of Molecular and Cell Biology, Henry Wellcome Building, University of Leicester, Leicester, UK; 2 LifeArc, Centre for Therapeutics Discovery, Open Innovation Campus, Stevenage, UK; 3 European Molecular Biology Laboratory, Grenoble, France

**Keywords:** single-domain antibody (sdAb, nanobody), cancer, phage display, immunoglobulin-like domain, immunotherapy, programmed death-ligand 1, programmed cell death protein 1, Cluster of differentiation 80, BLI, biolayer interferometry, CDR, complementary determining region, PDB, Protein Data Bank

## Abstract

Programmed death-ligand 1 (PD-L1) is a key immune regulatory protein that interacts with programmed cell death protein 1 (PD-1), leading to T-cell suppression. Whilst this interaction is key in self-tolerance, cancer cells evade the immune system by overexpressing PD-L1. Inhibition of the PD-1/PD-L1 pathway with standard monoclonal antibodies has proven a highly effective cancer treatment; however, single domain antibodies (VHH) may offer numerous potential benefits. Here, we report the identification and characterization of a diverse panel of 16 novel VHHs specific to PD-L1. The panel of VHHs demonstrate affinities of 0.7 nM to 5.1 μM and were able to completely inhibit PD-1 binding to PD-L1. The binding site for each VHH on PD-L1 was determined using NMR chemical shift perturbation mapping and revealed a common binding surface encompassing the PD-1–binding site. Additionally, we solved crystal structures of two representative VHHs in complex with PD-L1, which revealed unique binding modes. Similar NMR experiments were used to identify the binding site of CD80 on PD-L1, which is another immune response regulatory element and interacts with PD-L1 localized on the same cell surface. CD80 and PD-1 were revealed to share a highly overlapping binding site on PD-L1, with the panel of VHHs identified expected to inhibit CD80 binding. Comparison of the CD80 and PD-1 binding sites on PD-L1 enabled the identification of a potential antibody binding region able to confer specificity for the inhibition of PD-1 binding only, which may offer therapeutic benefits to counteract cancer cell evasion of the immune system.

The use of monoclonal antibodies (mAbs) to target the PD-1/PD-L1 immune signaling pathway has proven to be a highly successful immunotherapy for a range of cancers, with three approved mAbs targeting PD-L1 in clinical use and several currently under development (1). PD-1 (programmed cell death protein 1 or CD279) is a key regulator of the immune system and is expressed on the surface of T cells. Interaction of PD-1 with either cell surface PD-L1 (programmed cell death 1 ligand 1, B7-H1 or CD274) or PD-L2 (programmed cell death 1 ligand 2) induces the recruitment of SRC homology region 2 domain-containing phosphatases 1 and 2 (SHP-1 and SHP-2) to the intracellular domain of PD-1. This initiates a signal cascade leading to T-cell suppression (2, 3), which under normal conditions is a key regulator of immune self-tolerance. However, through the overexpression of cell surface PD-L1 (4), many types of cancer cells have co-opted this pathway as a means to avoid recognition and clearance by the immune system (4, 5).

PD-L1 is a 290 residue, type one transmembrane protein, which contains two extracellular domains: an IgV-like domain (PD-L1 D1) that binds to PD-1 and a membrane proximal IgC-like domain (PD-L1 D2) (6). PD-L2 shares a high sequence similarity to PD-L1 and binds to an overlapping binding site on PD-1 (7, 8). Inhibition of the PD-1/PD-L1 immune checkpoint pathway using mAbs against either PD-L1 or PD-1 has proven to be highly successful in treating a wide range of cancers (1); however, there remain some significant limitations including poor tumor penetration (9), off target toxicity (10), and declining patient response over time (11). More recently, there has been an increased interest in the use of single domain antibodies (VHHs) to target PD-L1. VHHs are single variable domain fragments of heavy chain–only antibodies derived from camelids. VHHs typically bind to their target antigens *via* the three complementary determining regions (CDRs) (12). Anti-PD-L1 VHHs have been used as part of bispecific antibody therapies (13, 14), as chimeric antigen receptor T-cell (CAR-T) therapies (15, 16), and a humanized anti-PD-L1 VHH is currently in phase III clinical trials (17).

CD80 (Cluster of differentiation 80, B7-1) is another functional partner of PD-L1 and also plays a key regulatory role within the immune synapse. In contrast to PD-1, which binds to PD-L1 displayed on another cell (in trans), CD80 forms heterodimers with PD-L1 on the same cell membrane (in cis) (18, 19). The formation of CD80/PD-L1 heterodimers inhibits PD-L1 binding to PD-1, as both CD80 and PD-1 share overlapping binding sites on PD-L1 (20). PD-L1 shows widespread expression in tumor cells, whereas CD80 is expressed primarily on antigen-presenting cells, including dendritic cells, and is upregulated after these cells are activated (21, 22, 23). Tumor infiltrating dendritic cells, expressing both PD-L1 and CD80, appear to play a key role in regulating T-cell antitumor response (24).

CD80 is a 254 residue type 1 transmembrane protein with a similar structure to PD-L1, consisting of an extracellular IgV-like domain that binds to both PD-L1 and it’s other functional partners CTLA-4 (cytotoxic T lymphocyte-associated protein 4) and CD28 (Cluster of differentiation 28), followed by a membrane proximal IgC-like domain (25). CD28 and CTLA-4 have opposing functions within immune regulation. CD28, upon binding to CD80, provides a stimulatory signal that leads to T-cell activation (26). CD28, despite being predominantly found as a dimer on the cell surface, interacts in a monovalent fashion with the CD80 monomer, dimer, and PD-L1/CD80 heterodimer (20, 27). In contrast to CD28, the recruitment of CTLA-4 to CD80 provides a downregulatory signal inhibiting T-cell activity. CTLA-4 is constitutively expressed on regulatory T cells (28, 29, 30) and on conventional T cells following activation (31, 32). CTLA-4 is believed to outcompete CD28 binding to CD80 due to its higher affinity and avidity (27), which leads to the formation of “zipper-like” lattices with dimeric CD80 that enhance T-cell downregulatory signaling (27, 33). CTLA-4 also depletes the level of available CD80 through *trans*-endocytosis (34) (Fig. 1).

**Figure 1:**
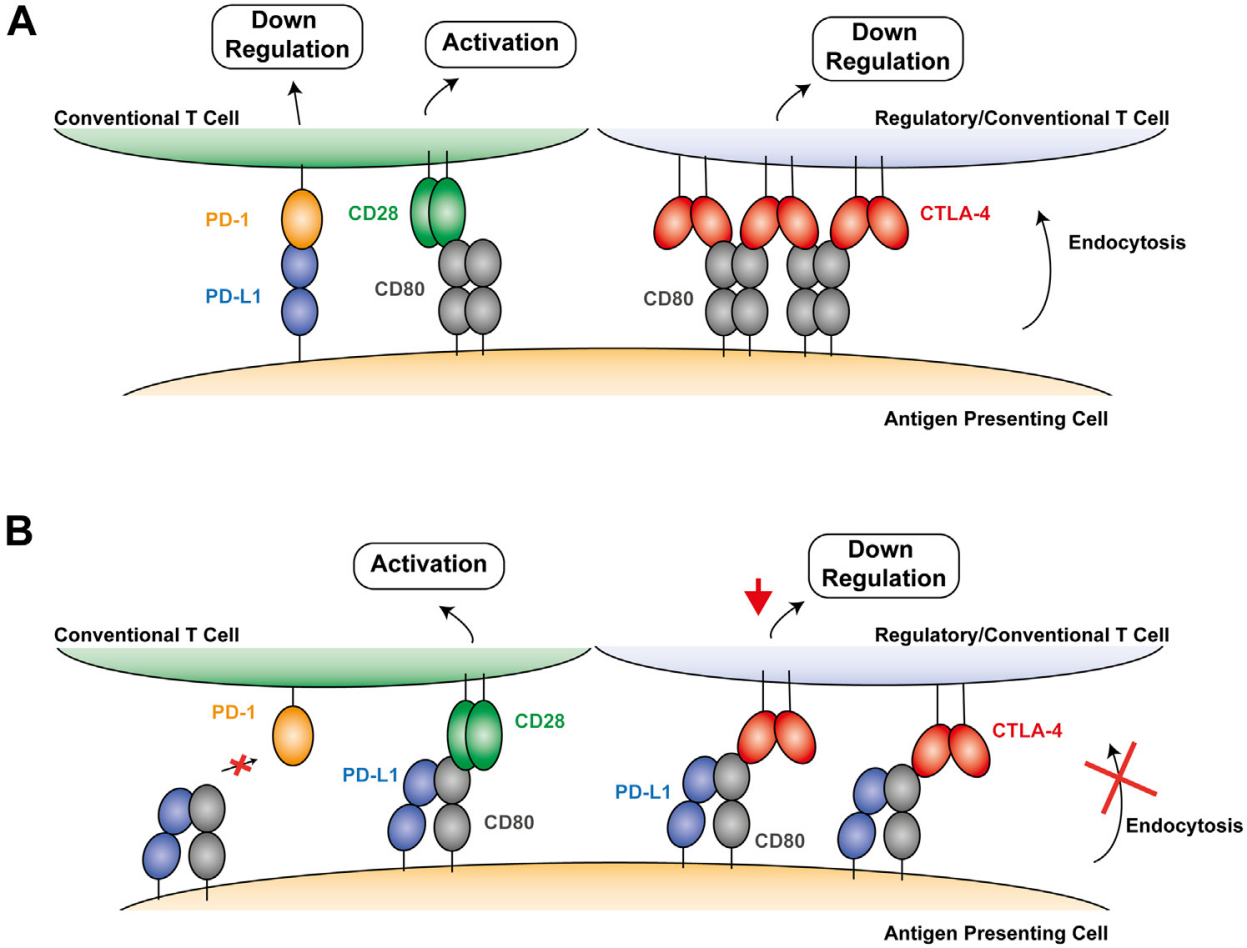
**Summary of PD-L1 and CD80 functional interactions.***A*, functional PD-L1 and CD80 interactions in the absence of PD-L1/CD80 heterodimers. Interaction of CD80 with CD28 induces T-cell activation (26). In contrast, PD-L1/PD-1 interaction mediates the downregulation of T-cell activity (2, 3). Similarly, CTLA-4 binds to dimeric CD80 to form ‘zipper-like’ lattices that enhance the downregulatory signaling of CTLA-4 (27, 33). CTLA-4 also depletes CD80 levels on antigen-presenting cells *via* endocytosis (34) *B*, summary of functional interactions with PD-L1/CD80 heterodimers. The PD-L1/CD80 heterodimer prevents PD-L1 from binding to PD-1, which prevents PD-1 induced downregulatory signaling of T cells but does not affect T-cell activation *via* CD28 binding to CD80 (20, 27). Formation of PD-L1/CD80 heterodimers decreases CTLA-4 mediated downregulation of activation by reducing the avidity of the CTLA-4/CD80 interaction and preventing the *trans*-endocytosis of CD80 (20).

The formation of PD-L1/CD80 heterodimers reduces CTLA-4 signaling by decreasing the avidity of the CTLA-4/CD80 interaction and preventing *trans*-endocytosis of CD80 (20). It has been suggested that the development of a therapeutic that was able to inhibit PD-L1 binding to PD-1, but not the interaction between CD80 and PD-L1, might have a greater therapeutic benefit than current therapies (20, 35, 36). Blocking the T-cell coinhibitory signaling through PD-1, whilst allowing the interaction of CD80 and PD-L1, would reduce the downregulatory signaling and depletion of CD80 by CTLA-4 but keep the T-cell costimulatory signaling of CD28. To date, all reported anti-PD-L1 mAbs that block binding of PD-1 have been shown to also inhibit the interaction with CD80. Potentially, the smaller binding site associated with VHHs could allow selective inhibition of PD-1 or CD80 binding to PD-L1.

In this communication, we report the identification and characterization of a diverse panel of 16 novel anti-PD-L1 VHHs, which show complete inhibition of PD-L1 binding to PD-1. The VHHs identified are specific to PD-L1 and all bind with nM to μM affinities. Surprisingly, NMR chemical shift perturbation mapping for these VHHs on PD-L1 revealed significantly overlapping epitopes within the PD-1–binding site. We have solved the crystal structures of two representative VHH/PD-L1 complexes, revealing distinct VHH binding modes to overlapping sites. Comparable NMR chemical shift perturbation mapping was also used to identify the binding site of CD80 on PD-L1, which revealed a highly overlapping binding site with PD-1. We also prepared an AlphaFold-Multimer model for the CD80/PD-L1 complex, which was produced prior to the publication of the crystal structure of PD-L1 bound to a high affinity variant of CD80 (37). This model shows close agreement with both the recently published crystal structure and the CD80-binding site determined by NMR. Overall, the work reported here reveals the surprising diversity of VHH antibody sequences able to recognize a key functional surface on PD-L1, together with pointing to the potential to selectively inhibit PD-1 binding to PD-L1 and not CD80.

## Results

### Identification of anti-PD-L1 VHHs

**Figure 2:**
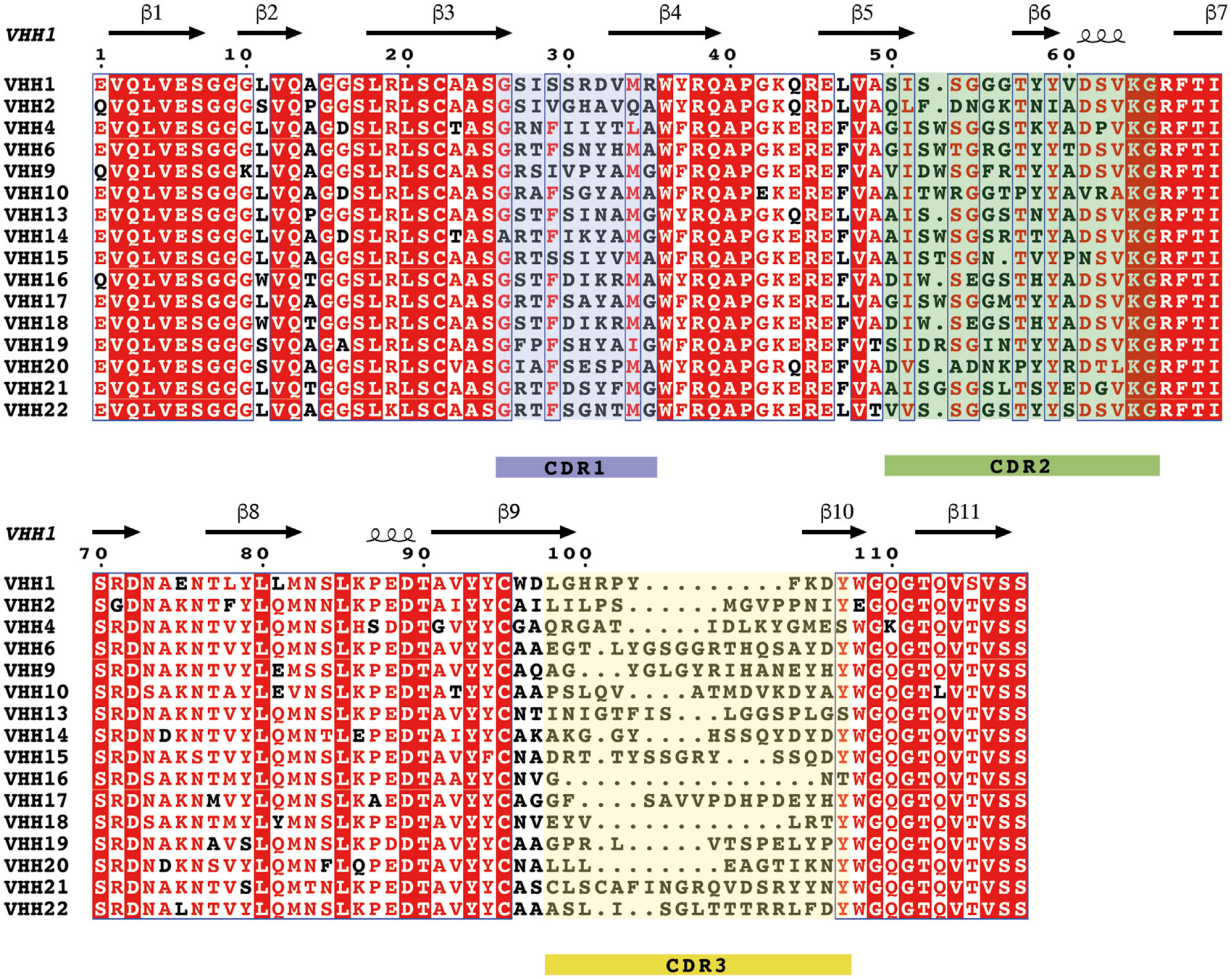
**Multiple sequence alignment of VHHs representative of the 16 families identified to bind to the D1 domain of PD-L1.** Sequences were aligned with Clustal Omega using the default Gonnet transition matrix and gap opening/extensions penalties of 6 and 1 (63). The figure was produced using ESPript (81), conserved residues are shown with a red background, with residues that are >70% conserved shown as *red* characters with *blue* frames. The VHH secondary structure shown above the alignment is for VHH1 bound to PD-L1. The numbers above the sequences correspond to VHH 1. CDRs are defined according to the Kabat numbering scheme (82) and are highlighted below the sequences. CDR, complementary determining region.

The extracellular region of PD-L1 D1D2 (residues 18–237) was panned against a naïve phage display VHH library (UCB BioPharma) prepared from 10 *Lama glama*. After two rounds of panning, individual clones were tested for binding to PD-L1 D1D2, the PD-1–binding domain of PDL1 (D1, residues 18–134), and the membrane proximal domain of PD-L1 (D2, residues 130–239). Clones found to bind PD-L1 were then sequenced and grouped into families based on sequence similarity. Eighty-five unique VHH sequences were identified that showed binding to PD-L1 D1D2, of which 27 sequences bound to PD-L1 D1, corresponding to 16 families, and 58 sequences bound to PD-L1 D2, corresponding to 27 families. VHHs that bound to PD-L1 D1 were chosen for further characterization as they were most likely to influence PD-L1 binding to PD-1. One representative sequence from each family that bound to PD-L1 D1 is shown in Figure 2. Lower sequence diversity was seen in CDR 1 and 2, compared to CDR 3. CDR 1 consists of 10 residues for all sequences, which formed four distinct CDR families. CDR 2 comprises of 16 to 17 residues with eight distinct families. Two VHHs (16 and 18) had identical sequences for CDR 1 and 2 but a distinct CDR 3 sequence. In contrast, the 16 representative CDR 3 sequences ranged from 3 to 19 residues in length. Interestingly, VHH21 has two cysteine residues within CDR 3, separated by two amino acids, with the formation of an additional disulfide bond confirmed by mass spectrometry.

### Characterization of anti-PD-L1 D1 VHHs

**Figure 3:**
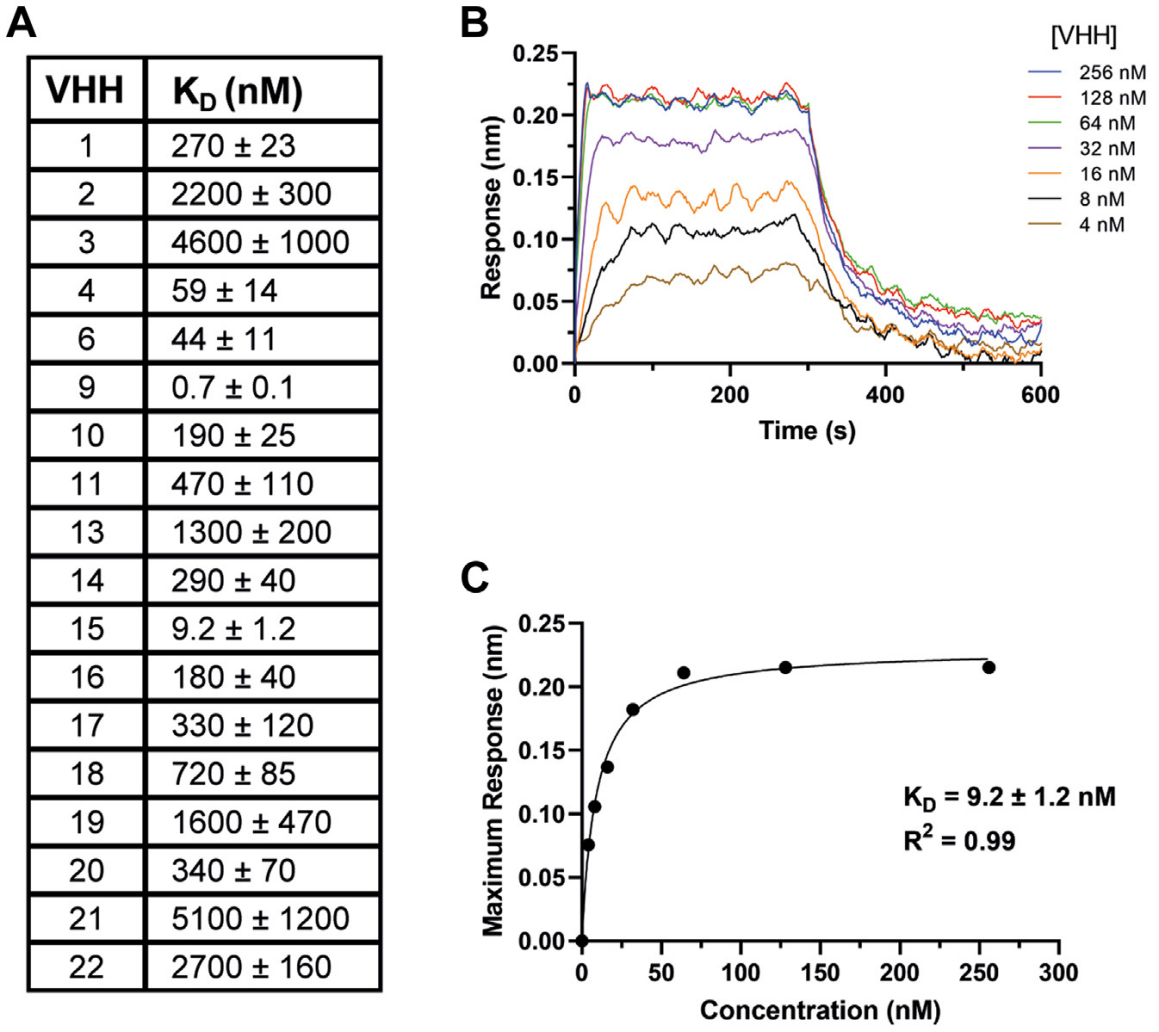
**Determining the affinity of representative VHHs binding to PD-L1.***A*, a summary of the *K*_*D*_ values determined for the representative set of anti-PD-L1 VHHs by BLI. Errors included are the SEM calculated for individual fitted steady state *curves* using Prism version 9. *B*, a typical BLI sensorgram obtained for a VHH binding (VHH15) to immobilized PD-L1-Fc, with the association and dissociation *curves* shown for a range of VHH concentrations. *C*, the steady state binding *curve* derived from the maximum response observed for VHH15. The *K*_*D*_ was obtained by fitting the maximum response to a one-site specific-binding model (Prism version 9).

To further characterize the panel of PD-L1 D1 binding VHHs, *Escherichia coli* expression vectors were prepared with a tobacco etch virus (TEV) protease cleavable N-terminal hexa-histidine tag. The expressed VHHs were refolded and purified as described previously (38), providing yields of ∼8 to 30 mg/l of purified VHH. Biolayer interferometry (BLI) was used to determine the affinity (*K*_*D*_) for each VHH binding to PD-L1, using a PD-L1-Fc fusion protein (Invitrogen) immobilized on Protein G biosensors (Sartorius). *K*_*D*_ values were determined by steady-state equilibrium analysis of the maximum sensorgram responses observed over a range of VHH concentrations (Fig. 3). The VHHs displayed a wide range of affinities ranging from 0.7 nM to 5.1 μM, with 12 having a *K*_*D*_ of <1 μM (Fig. 3). Additional kinetic analysis was carried out to determine k_on_, k_off_, and *K*_*D*_ values for selected high affinity VHHs, which produced *K*_*D*_s in close agreement with the values determined using steady state analysis (Table S1).

**Figure 4:**
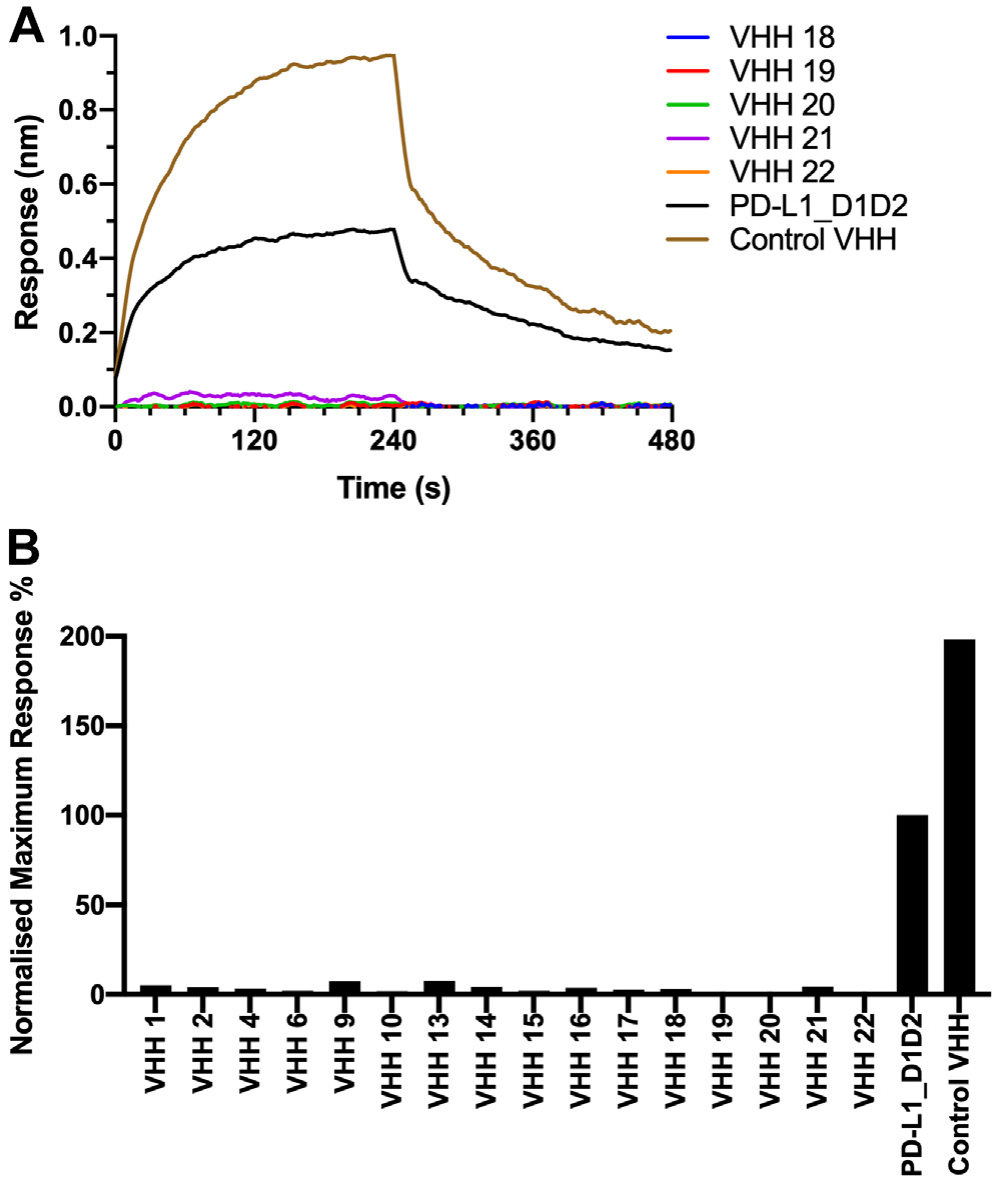
**A diverse panel of VHHs inhibit PD-L1 binding to PD-1 in BLI experiments.** The maximum response observed for PD-L1 D1D2 (3 μM) binding to immobilized PD-1 in the presence of a high concentration of VHH (20 μM) was compared to the response in the absence of the VHH. This concentration was saturating for the majority of the VHHs (10/16) with the weakest VHH expected to bind to 80% of the free PD-L1. An increase in the maximum response observed indicates that the PD-L1 D1D2⋅VHH complex is binding to PD-1, whereas a decrease in the maximum response seen reveals that the VHH is inhibiting the binding of PD-L1 to PD-1. *A*, representative sensorgrams showing the responses observed for VHH 18 to 22 in complex with PD-L1. Traces for PD-L1 D1D2 alone and a noninhibitory VHH that binds to PD-L1 D2 are also shown. Only the control PD-L1 D2 binding VHH shows an increase in maximum response compared to PD-L1 D1D2 alone, with all the other VHHs showing a complete inhibition of PD-L1 binding to PD-1. *B*, for the panel of VHHs, the maximum BLI response observed is shown as a percentage of PD-L1 D1D2 alone binding to PD-1. All the anti-PD-L1 D1 VHHs showed a greater than 90% inhibition of PD-L1 binding to PD-1. BLI, biolayer interferometry.

BLI was similarly used to determine whether the VHHs bound to PD-L2, with the extracellular region of PD-L2 immobilized on Protein G biosensors with a Fc tag (Generon); however, none of the VHHs showed any evidence of binding to PD-L2. The panel of VHHs were also assessed for their ability to inhibit PD-1 binding using a BLI assay, in which avi-tagged PD-1 was immobilized on streptavidin biosensors (Sartorius) and incubated with PD-L1 D1D2 (3 μM) in the presence of a nearly 6.7-fold molar excess (20 μM) of each VHH (Fig. 4). Somewhat surprisingly, all the VHHs completely inhibited PD-L1 binding to PD-1.

### Determination of the epitopes for the panel of PD-L1 D1 binding VHHs

**Figure 5:**
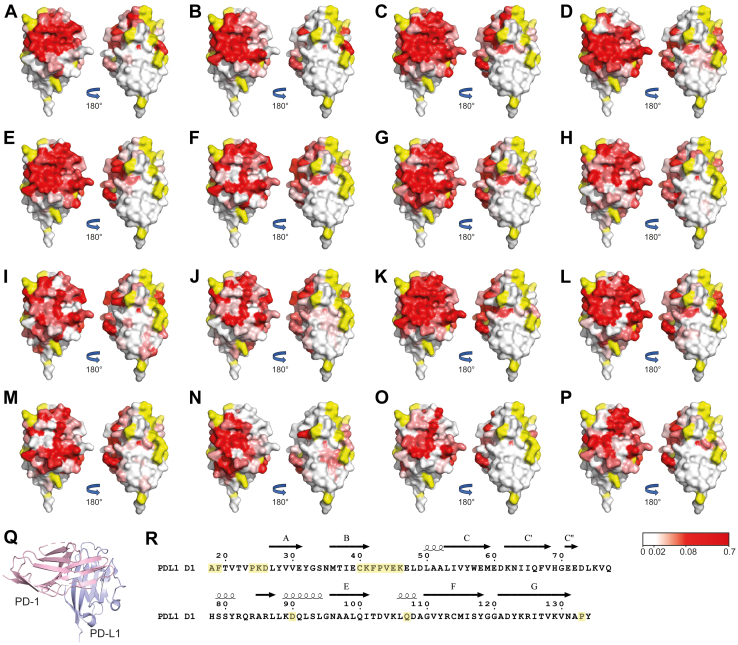
**Mapping of VHH interaction sites on PD-L1 D1 by NMR minimal shift analysis of backbone signals.***A*–*P*, the backbone minimal shift for each residue was obtained by comparing the VHH bound and free TROSY or HNCO spectra of PD-L1 D1. The minimal shifts obtained have been mapped onto the surface of the reported PD-L1 D1 structure (PDB: 5C3T (6)). Residues are colored based on the magnitude of the backbone minimal shifts seen, with residues showing a minimal shift of ≤0.02 ppm shown in white, 0.02 to 0.08 ppm colored in a linear gradient from *white* to *red* and ≥0.08 ppm shown in *red*. Residues for which no minimal shift data could be obtained are colored in *yellow*. The minimal shift surface view of PD-L1 D1 is shown for binding of (*A*) VHH1, (*B*) VHH2, (*C*) VHH4, (*D*) VHH6, (*E*) VHH9, (*F*) VHH10, (*G*) VHH13, (*H*) VHH14, (*I*) VHH15, (*J*) VHH16, (*K*) VHH17, (*L*) VHH18, (*M*) VHH19, (*N*) VHH20, (*O*) VHH21, and (*P*) VHH22. In (*Q*), a ribbon representation of the PD-1⋅PD-L1 complex (PDB: 4ZQK (6)), with PD-1 in *pink* and PD-L1 D1 in *blue* is shown in the same orientation as (*A*–*P*). The sequence of human PD-L1 D1 is shown in (*R*) with the position of elements of regular secondary structure displayed above the sequence (42) and unassigned residues highlighted in *yellow*. This panel was produced with ESPript (81). PDB, Protein Data Bank.

To identify the binding sites for the panel of VHHs on PD-L1 D1, NMR chemical shift perturbation mapping was used. We have previously reported sequence-specific ^15^N, ^13^C, and ^1^H backbone NMR assignments for PD-L1 D1 (39), which enabled 2D ^15^N/^1^H transverse relaxation optimized spectroscopy (TROSY) and/or ^15^N/^13^C/^1^H HNCO spectra to be collected for PD-L1 D1 in complex with each inhibitory VHH and compared to the spectra of free PD-L1 D1 (Fig. S1). Minimal shift analysis of the spectra was used to identify backbone amide-associated peaks that showed a significant change in position due to either direct interaction with a VHH or due to induced conformational change, as described previously (40). The observed chemical shift perturbations for backbone amide associated peaks were mapped onto the reported PD-L1 D1 crystal structure (Protein Data Bank [PDB]:5C3T (6)) for each of the VHHs. This revealed a fairly contiguous surface affected by antibody binding (Fig. 5) with an average apparent binding surface area of 900 ± 290 Å^2^. Typical VHH epitopes have been reported to correspond to a buried surface area of 750 ± 180 Å^2^, which corresponds to 13 ± 6 residues usually involved in interactions with nanobodies (41). The diverse panel of VHHs all induced large shifts in backbone NMR signals from PD-L1 residues that form part of the PD-1–binding site (42), implying that they sterically block PD-1 binding. Surprisingly, no VHHs were identified that bound to the opposite face of PD-L1 D1 to that involved in PD-1 binding (Fig. 5). In addition, all of the VHHs caused substantial shifts in signals from residues within the C′ β-strand of PD-L1 D1, (residues 54–60). This region is not present in PD-L2, which potentially explains the specificity of the inhibitory VHHs to PD-L1, as shown in the BLI experiments presented here.

### Crystal structures of representative PD-L1⋅VHH complexes

**Figure 6:**
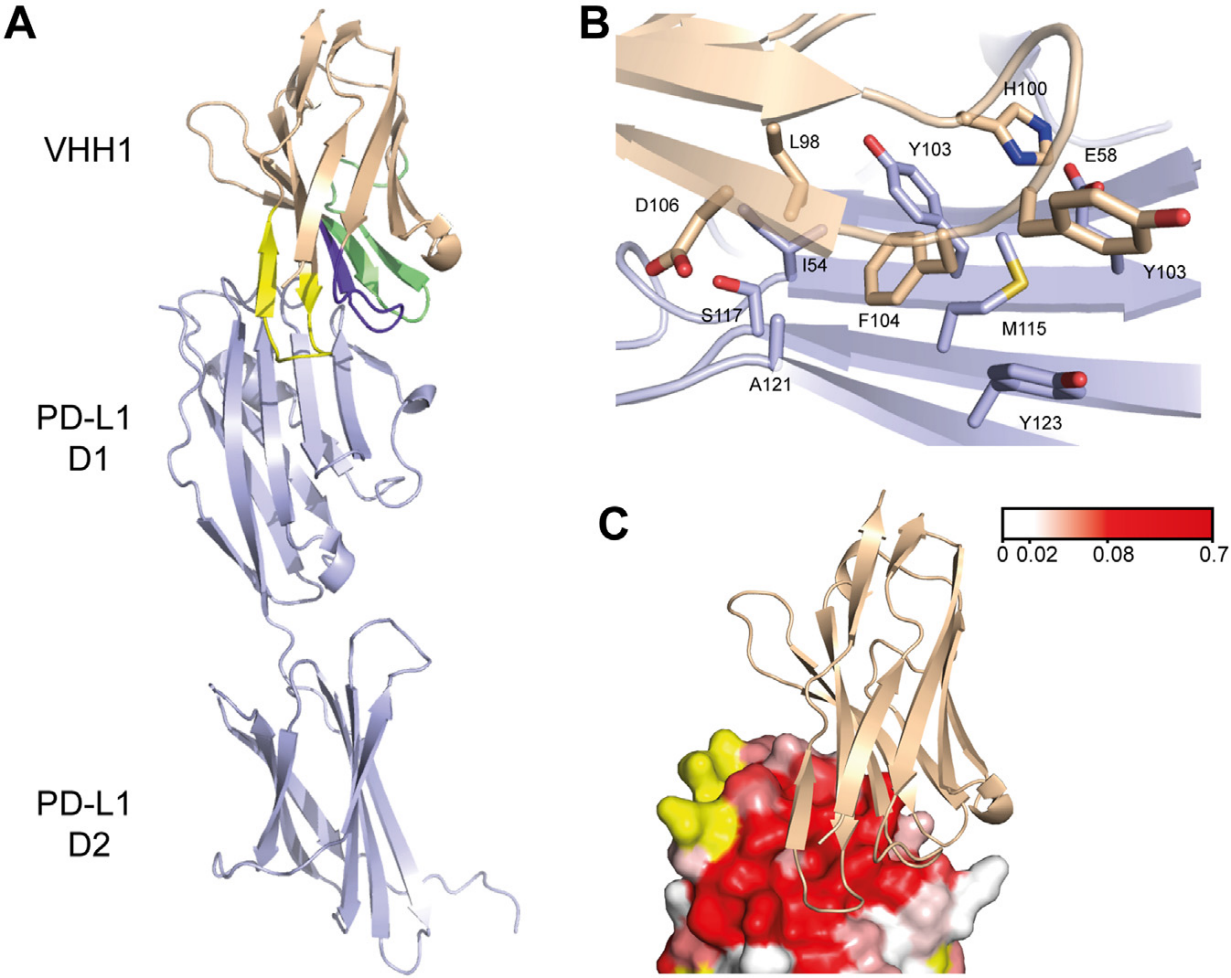
**Crystal structure of the VHH1⋅PD-L1 D1D2 complex.** PD-L1 is displayed in *light blue* and VHH1 in *light orange*. *A*, ribbon representation of VHH1 binding to PD-L1 D1D2 with CDR 1, 2, and 3 colored in *purple*, *green*, and *yellow*, respectively. *B*, a view of the interface centered on F104 from VHH1, which sits within a hydrophobic pocket of PD-L1 D1. *C*, a surface view of the VHH-binding site on PD-L1 D1 with VHH1 (*light orange*) shown as a ribbon representation and PD-L1 D1 shown as a solvent accessible surface colored on the basis of the backbone amide minimal shifts induced by VHH1 binding. CDR, complementary determining region.

**Figure 7:**
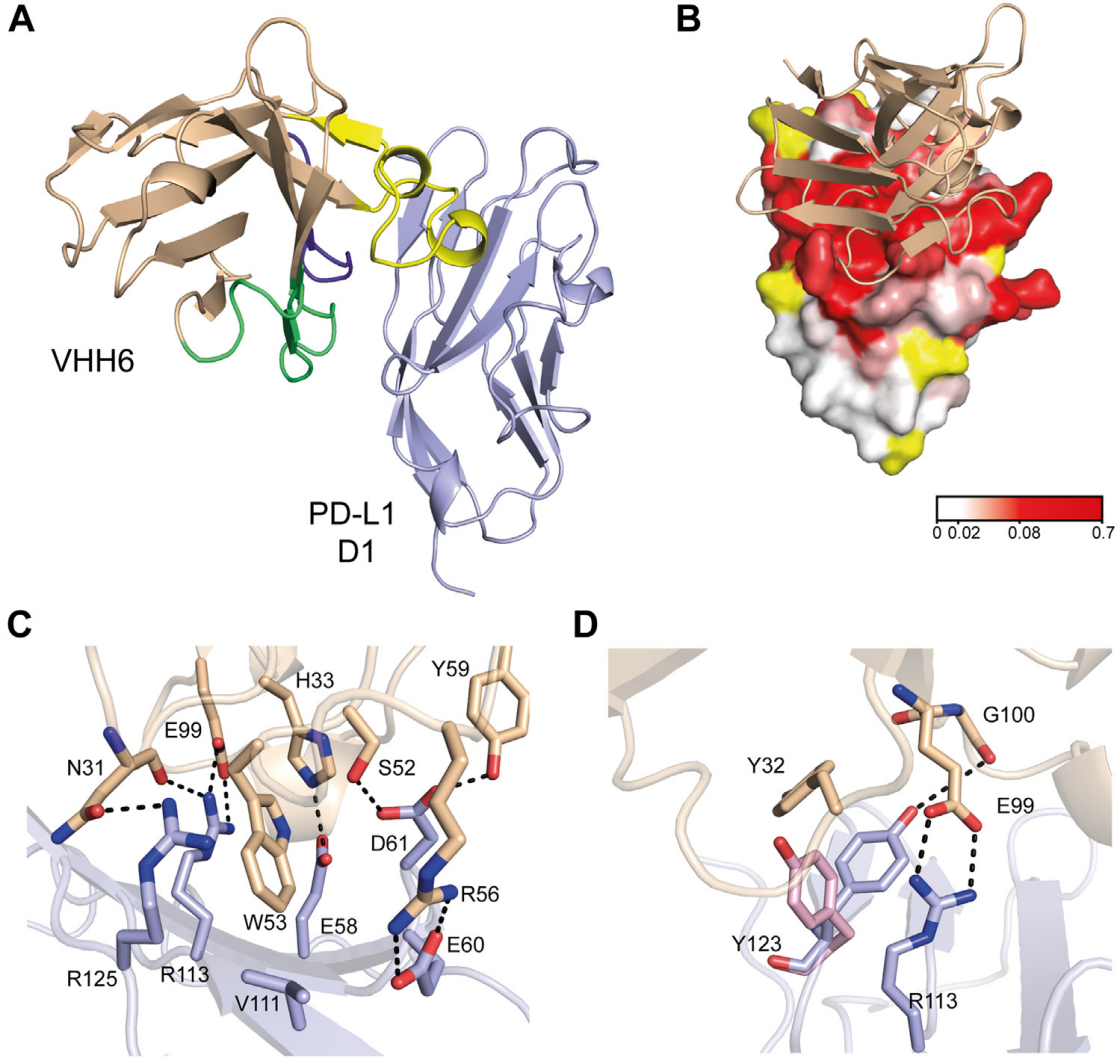
**Crystal structure of the VHH6⋅PD-L1 D1 complex.** PD-L1 is displayed in *light blue* and VHH6 in *light orange*. *A*, a ribbon representation of VHH6 binding to PD-L1 D1D2 with CDR 1, 2, and 3 colored in *purple*, *green*, and *yellow*, respectively. *B*, a surface view of the VHH6-binding site on PD-L1 D1, which is colored according to the backbone NMR minimal shifts induced by binding of the VHH. The position of the bound VHH6 is shown as a ribbon representation. *C*, binding pocket on PD-L1 D1 for W53 from VHH6. W53 binds in a pocket on PD-L1 D1, which is facilitated by a network of intermolecular bonds from surrounding residues. *D*, conformation of Y123 of PD-L1 when bound to VHH6. The PD-1 bound placement of Y123 is shown in *pink* (PDB: 4ZQK (6)). The hydrogen bond between G100 of the VHH and PD-L1 Y123 holds Y123 in a “closed” conformation. CDR, complementary determining region.

To further characterize the interaction of the inhibitory VHHs with PD-L1, crystal structures were solved for representative VHHs (VHH1 bound to PD-L1 D1D2 and VHH6 bound to PD-L1 D1). As expected, VHH1 and 6 adopt the classical fold of a VHH (43), with four framework regions consisting mainly of β-strands and a conserved disulfide bond between framework regions 1 and 3. As described in detail later, residues within the CDRs were seen to form the majority of the contacts with PD-L1 D1. The overall structure of PD-L1 D1 was very similar in both complexes, with an average RMSD for Cα positions to free PD-L1 D1 (PDB: 3BIS (42)) of 0.6 Å for both VHH1 and 6. The crystal structures obtained were entirely consistent with the VHH binding interfaces determined by the minimal shift analysis of NMR spectra, as shown in Figures 6*C* and 7*B*.

### VHH1⋅PD-L1 D1D2 complex structure

The crystal structure of VHH1 bound to PD-L1 D1D2 (Fig. 6) was determined to 2.2 Å and revealed one complex molecule in the asymmetric unit. For VHH1, residues 1 to 119 were well defined and could be modeled into the electron density maps calculated. For PD-L1, residues 18 to 235 were modeled, except residues 215 to 217, which lie within a flexible surface loop of D2. PISA analysis of the crystal structure of the complex revealed that the PD-L1 D1⋅VHH1 interface contains a combined buried surface area of 1178 Å^2^, with 600 Å^2^ from PD-L1 and 578 Å^2^ from VHH1. Residues within the CDR regions contribute 95% of the total buried VHH surface area, with CDR 3 showing a 32% reduction in solvent accessibility. Major changes were also seen in CDR 1 and CDR 2 (28% and 19% reductions, respectively). The interaction between VHH1 and PD-L1 D1D2 includes four hydrogen bonds, R35 within CDR 1 to A51, S50 within CDR 2 to H69, S53 within CDR 2 to Q66, and H100 in CDR 3 to E58. Another key interaction involves F104 in CDR 3, which sits in an induced hydrophobic pocket on PD-L1 D1, with I54, S117, and A121 forming the top of the pocket and M115, Y123, and Y56 forming the bottom (Fig. 6*B*). This region has previously been identified as one of three druggable hot spots on the surface of PD-L1 D1 that would be favorable in accommodating an aromatic ring (6).

### VHH6⋅PD-L1 D1 complex structure

The crystal structure of VHH6 bound to PD-L1 D1 (Fig. 7) was solved to a resolution of 1.6 Å and also contained one molecule of the complex in the asymmetric unit. For the VHH, all residues were visible in the electron density map and able to be modeled, while for PD-L1, residues 18 to 132 were able to be modeled. The complex was found to contain a total buried surface area of 1449 Å^2^ with 727 Å^2^ for VHH6 and 722 Å^2^ for PD-L1 D1. All of the residues from VHH6 involved in interactions with PD-L1 are located in the three CDR loops. Specifically, CDR 1 loses 38% of its solvent accessible area upon PD-L1 binding, whereas CDR 2 and CDR 3 lose 25% and 31%, respectively. The contact surface features a network of polar and charge interactions, with each CDR making a salt bridge to PD-L1 D1. CDR 2 is involved in two hydrogen bonds, whereas both CDR 1 and CDR 3 are involved in three. W53 from CDR 2 is a key residue losing almost all solvent accessibility and sitting in a pocket on PD-L1 formed by residues E58, E60, D61, R113, and R125 (Fig. 7*C*). This pocket also accommodates an aromatic ring in both the durvalumab (44) and atezolizumab (45) bound PD-L1 structures. The large network of polar and charged interactions underlies the high affinity of VHH6 for PD-L1, with a *K*_*D*_ of 49 nM. This structure reveals an interesting conformation of Y123 within PD-L1, as VHH 6 stabilizes the side chain in a “closed conformation,” resulting in it pointing inward toward the β-sheet of PD-L1 (Fig. 7*D*). This conformation is not present in any of the previously published antibody or nanobody bound PD-L1 structures (13, 44, 45, 46, 47), PD-1 bound (6) or apo structures (42). In these complexes, Y123 is in an “open” conformation, which in the PD-L1⋅PD-1 complex structure allows a pocket to form in which I134 residue of PD-1 can interact, whereas in our complex Y123 would clash with I134 from PD-1.

### Molecular basis of the inhibition of PD-1 binding to PD-L1 by VHH1 and 6

We compared the crystal structures of VHH1 and VHH6 bound to PD-L1 to the previously reported PD-1/PD-L1 structure (6) to elucidate how the VHHs inhibit PD-1 binding (Fig. S3). For VHH1, CDR1, CDR3, and the N terminus of the VHH sterically clash with PD-1 bound to PD-L1 D1. CDR 3 clashes with the C strand and FG loop of PD-1 and CDR 1 interacts where the FG loop of PD-1 interacts with PD-L1. VHH6 shows a greater overlapping binding site with PD-1 when compared to VHH1. VHH6 contacts nearly all of the PD-L1 residues that are involved in PD-1 binding, with the exception of those interacting with the C’’D loop of PD-1. Steric blocking of the PD-1–binding sites, together with the higher affinity for PD-L1, explains why both VHHs completely inhibit PD-1 binding to PD-L1.

### Identification of the CD80-binding site on PD-L1 D1

**Figure 8:**
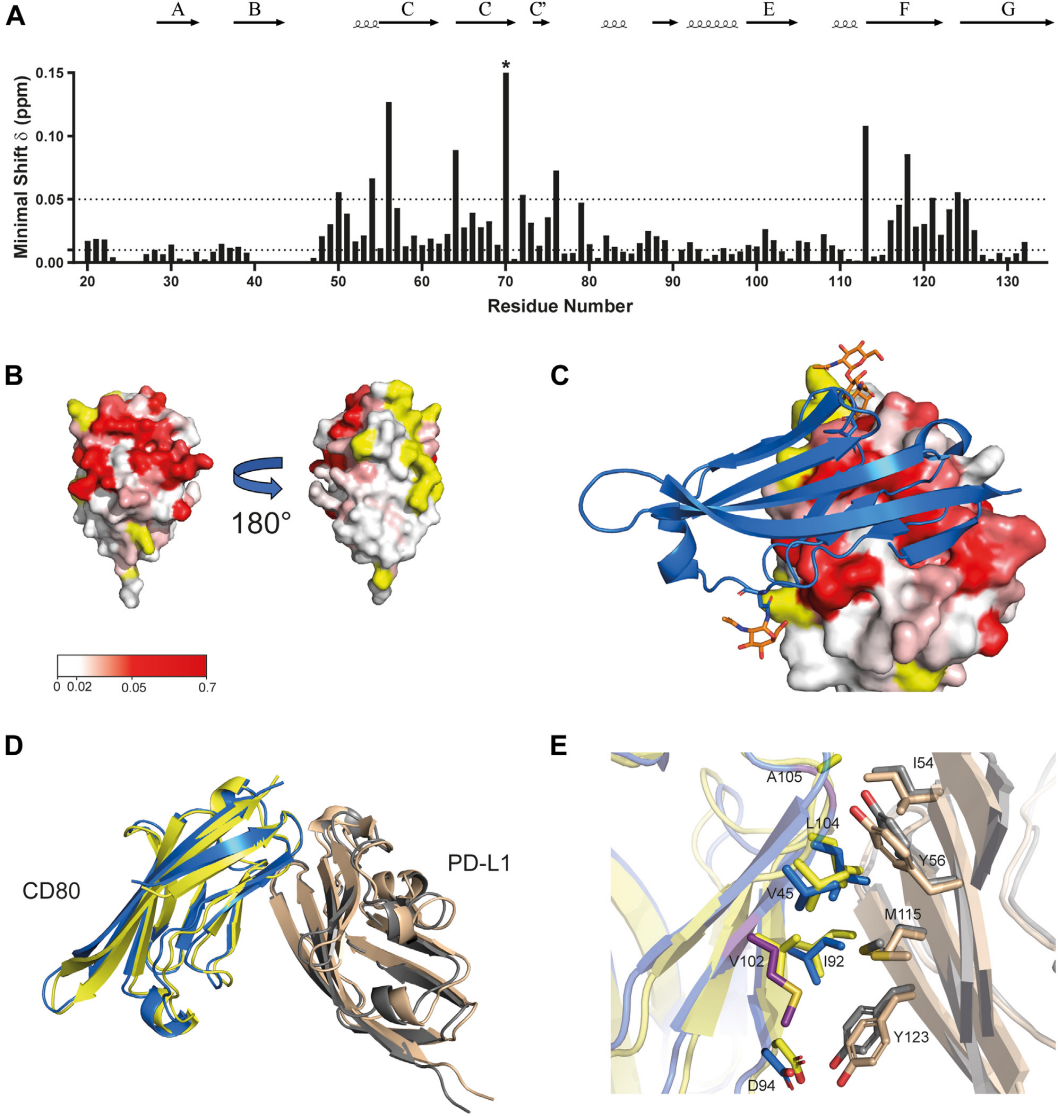
**Identification of the CD80-binding site on PD-L1 D1 by NMR minimal shift analysis.***A*, a histogram displaying the backbone amide minimal shifts (^15^N and ^1^H) observed for individual residues of PD-L1 D1 on binding CD80 (∗ residue shows a much greater minimal shift of 0.64 ppm). *B*, surface views of PD-L1 with the minimal shifts observed for backbone amides on CD80 binding shown (PDB: 5C3T (6)). Residues are colored on the basis of the magnitude of the backbone amide minimal shifts induced by CD80 binding, with residues showing a minimal shift of ≤0.01 ppm shown in *white*, 0.01 to 0.05 ppm colored in a linear gradient from *white* to *red* and ≥ 0.05 ppm shown in *red*. Residues where no minimal shift data could be obtained are colored in *yellow*. *C*, the binding interface revealed in the structure of the CD80 IgV variant⋅PD-L1 D1D2 complex structure (PDB: 7TPS (37)). The CD80 IgV domain (*blue*) is shown as a ribbon representation with selected glycosylation sites highlighted (*orange*). PD-L1 D1 is shown as a solvent accessible surface colored on the basis of the backbone amide minimal shifts induced by CD80 binding, as indicated in panel (*B*). *D*, superposition of the interacting domains from the PD-L1 D1D2⋅CD80 IgV complex solved by X-ray crystallography (*orange*/*blue*) (PDB: 7TPU) and predicted by AlphaFold (*gray*/*yellow*). The alignment was done on the basis of all Cα atoms and revealed an average RMSD of 1.3 Å. *E*, comparison of the PD-L1⋅CD80 binding interface seen in the AlphaFold model and reported crystal structure, with the protein domains colored as depicted in panel (*D*). The side chains of key residues involved in intermolecular contacts are shown and labeled. Interface residue V102 of CD80 is mutated to methionine in the high affinity CD80 IgV variant and is shown in *purple*. PDB, Protein Data Bank.

To map the CD80-binding surface of PD-L1, the extracellular region of CD80 was expressed in CHO cells using the native signal sequence. ^15^N/^1^H TROSY spectra for ^15^N-labeled PD-L1 D1 bound to CD80 were obtained as previously described for mapping the VHH interaction sites, with a 60% molar excess of CD80 present in the NMR sample (Fig. S2). Backbone amide peaks of PD-L1 D1 that showed significant chemical shift perturbations by CD80 binding were again identified by minimal shift analysis (Fig. 8*A*). Previous mutational studies have suggested that residues D49, I54, Y56, E58, N63, R113, M115, G119, and K124 are involved in PD-L1 binding to CD80 (19, 35). Backbone amide peaks from all these residues showed significant shifts upon CD80 binding with the exception of M115, which is in a region of the ^15^N/^1^H TROSY spectra that has many overlapping signals, so perturbation of this signal would be underestimated. Furthermore, residues E58, N63, and V76 of PD-L1 (19) have been reported to have no influence on CD80 binding, which is consistent with no significant shifts observed in the corresponding backbone amide NMR peaks. Mapping of the chemical shift perturbations induced by the binding of CD80 on the surface of PD-L1 D1 shows a considerable overlap in the binding sites of PD-1 and CD80. Comparison with the chemical shift perturbations induced by VHH binding to PD-L1 also shows considerable overlap with the CD80-binding site, indicating that none of the VHHs are likely to specifically inhibit PD-1 binding without affecting the interaction with CD80 (Figs. 5 and 8). CD80 binding also causes substantial shifts in backbone amide peaks from residues in the C′ β-strand, which is not present in PD-L2. This contrasts to PD-1, which makes minimal interactions with the C′ β-strand of PD-L1 (18) and may explain the specificity of CD80 to PD-L1.

### AlphaFold model of PD-L1 binding to CD80

AlphaFold-Multimer (48, 49) was used to obtain a predicted structure for the PD-L1⋅CD80 complex and the reliability of the top-ranked model was assessed using the NMR determined CD80-binding site on PD-L1. The amino acid sequences for the extracellular regions of PD-L1 D1D2 and CD80 were used as input for AlphaFold-Multimer, which produced a top-ranked model with a high confidence score (0.86). The AlphaFold-Multimer model predicted the same binding site on PD-L1 for CD80 as revealed by the NMR chemical shift perturbation results reported here. The predicted binding site for CD80 also agrees with published mutational studies, showing that both I92R/E and L104E inhibited CD80 binding to PD-L1, with both residues located at the center of the predicted CD80-binding site (35). CD80 has two other functional binding partners, CTLA-4 and CD28, which have been shown to have overlapping binding sites on CD80 (33, 50, 51) that do not block PD-L1 binding (20). This is consistent with the PD-L1⋅CD80 complex model and NMR data, which places the PD-L1 binding site on the opposite face to that involved in CTLA-4/CD28 binding. Furthermore, the Y56A mutation on PD-L1 has been shown to disrupt CD80, but not PD-1 binding (35), which is consistent with a hydrogen bond interaction seen between Y56 and the backbone of CD80 (K43) in the AlphaFold-Multimer model, whereas Y56 plays no role in the PD-1/PD-L1 interface (6).

During the preparation of this article, a structure of PD-L1 D1D2 in complex with a high affinity variant of the CD80 IgV domain was reported (37). This CD80 variant contained seven single residue substitutions, with two located within the PD-L1–binding site. Figure 8*C* shows an overlap of the reported PD-L1⋅CD80 IgV domain complex structure, with the NMR backbone amide minimal shifts induced on PD-L1 D1 by CD80 binding. The CD80-binding site determined by NMR overlays almost perfectly with the contact surface observed in the CD80 IgV domain variant. In both our NMR experiments and the recently reported X-ray structure, glycosylated forms of CD80 were used. The oligosaccharide chain attached to N89 makes several specific contacts with PD-L1 D1, including potential hydrogen bonds with Y118 and D49, both of which show large minimal shifts in our NMR experiments. The similarity between the reported X-ray structure of PD-L1 D1D2⋅CD80 IgV and our predicted AlphaFold-Multimer model (Fig. 8, *D* and *E*) was striking. Specifically, the model correctly predicted 85% of the native contacts between PD-L1 and CD80, which is reflected in an interface backbone RMSD of 1.0 Å. Of the six intermolecular hydrogen bonds observed in the X-ray structure of the complex, AlphaFold-Multimer correctly predicted three, including the hydrogen bonds between PD-L1 Y56 and CD80 K43. The three intermolecular hydrogen bonds not predicted by AlphaFold-Multimer lie within a flexible loop region of CD80. The comparison reported here demonstrates how remarkably accurate AlphaFold-Multimer can be at predicting protein–protein complexes, including fine detail at the protein–protein interface.

## Discussion

The work reported here has led to the identification of a diverse panel of anti-PD-L1 D1 VHHs, consisting of 16 families with affinities ranging from 0.7 nM to 5.1 μM. Judging from the sequence diversity and range of affinities observed for the panel of VHHs, a range of different epitopes were expected. Surprisingly, all of the VHHs targeted a common surface region of PD-L1 D1 that encompasses the PD-1–binding site, enabling complete inhibition of PD-1 binding. The reason for this bias in the surface region recognized is not clear. One possibility is a presentation bias during panning, conceivably due to PD-L1 interaction with plastic plates resulting in only the PD-1–binding site being accessible. Alternatively, the PD-1–binding site as a functional protein–protein interface may be the most attractive interface for antibody interactions. Although, this single binding surface preference was not seen for anti PD-L1 D2 antibodies identified by the same panning procedure (unpublished findings). Interestingly, all the reported antibodies targeting PD-L1 D1 have been shown to block PD-1 binding; however, this could reflect a selective preference to identify PD-1 inhibitory antibodies. Despite the growing number of inhibitory anti-PD-L1 VHHs (13, 47, 52, 53), these tend to be skewed toward VHHs with high affinities (low nM) and there remain few with fully characterized epitopes. To date, there are only two reported structures of VHH⋅PD-L1 complexes (13, 47). Here, we report the structures of two distinct VHHs bound to PD-L1, which reveal unique binding modes when compared to each other and the previously published anti-PD-L1 VHH structures.

Targeted inhibition of PD-L1 binding to PD-1 without blocking the interaction with CD80 has been proposed to have significant potential therapeutic benefits (20, 35, 36). A therapeutic that inhibits PD-L1 binding to PD-1 but not PD-L1/CD80 heterodimer formation would, alongside the benefit of inhibiting the PD-L1/PD-1 pathway, allow CD80 to upregulate the T-cell response through CD28 signaling (26), whilst reducing the T-cell coinhibitory effects of CTLA-4 binding to CD80 (27, 33, 34) (Fig. 1). Previous attempts to generate antibodies specifically targeting the ability of PD-L1 to bind PD-1 over CD80 have been unsuccessful (19); however, there is one reported antibody that appeared to preferentially inhibit the CD80/PD-L1 interaction (24). To assess the feasibility of selectively targeting the PD-L1/PD-1 over the PD-L1/CD80 interaction, we used NMR to identify the binding site for CD80 on PD-L1, which revealed an identical binding site to that seen for the high affinity CD80 IgV variant bound to PD-L1 in a recently reported crystal structure (37). The reported PD-L1/CD80 IgV variant structure showed very close agreement with our AlphaFold-Multimer model generated for the PD-L1/CD80 complex, which is a striking illustration of the potential of AlphaFold-Multimer to provide accurate predictions for protein–protein complexes. The substantial overlap between the epitopes of the 16 inhibitory anti-PD-L1 VHHs reported here and the CD80-binding site revealed that none would be able to selectively inhibit PD-1 binding over CD80.

PD-1 and CD80 share a heavily overlapping binding site on PD-L1 (Fig. S3); however, comparison of the crystal structures reported for PD-L1 bound to the CD80 IgV variant (37) and PD-1 (6) revealed a potential avenue to selectively inhibit PD-1 binding. Six residues from PD-L1 (A18, F19, T20, D26, K124 and R125) make exclusive contacts with PD-1, with residues F19, T26, and D125 forming hydrogen bonds with PD-1 and K124 forming a salt bridge. This region on PD-L1 has previously been identified as a hot spot for the PD-1/PD-L1 interaction (54) and makes contacts with the C_C’’ loop region of PD-1. The importance of the C-C’’ region on PD-1 is supported by mutational studies revealing residues M70, T76, and K78 as key in stabilizing (55, 56, 57) the PD-1/PD-L1 interaction. In contrast, selective targeting of the CD80/PD-L1 interaction could be achieved by targeting the C and C′ strands of PD-L1. Antibodies that bind here would sterically clash with the IgC-like domain of CD80 that follows the IgV domain. Due to the location of the CD80-binding interface, it is likely that the IgC-like domain would make some, though not essential (35), contacts with PD-L1. This is demonstrated in our AlphaFold-Multimer model where E194, within the IgC-like domain of CD80, makes contacts with PD-L1. In conclusion, we have identified potential new opportunities for selectively targeting PD-L1 binding to PD-1 without the inhibition of CD80 binding, which may provide the basis for new and improved therapeutic antibodies. However, this will require the generation and careful selection of a new panel of anti-PD-L1 D1 VHHs.

## Experimental procedures

### Expression and purification of the extracellular regions of PD-L1 and PD-1

Constructs encoding the extracellular regions of human PD-L1 D1D2 (18–237), PD-L D1 (18–134), and PD-1 (33–150) with a C-terminal avitag (GGLNDIFEAQKIEWHE) were synthesized and cloned into pET28a by GenScript. A C-terminal avitag construct of PD-L1 D1D2 (18–237) was produced by mutagenesis (https://openwetware.org/wiki/%27Round-the-horn_site-directed_mutagenesis, accessed February 2, 2022) (58). PD-L1 D2 (130–239) was cloned into the pLEICS-05 vector by PROTEX (University of Leicester). PD-L1 and PD-1 constructs were expressed and refolded as previously reported (6). Proteins were purified using a Superdex 75 26/600 gel filtration column (GE Healthcare) equilibrated in 25 mM KH_2_PO_4_, pH 7.4, 25 mM NaCl, and 10 μM EDTA. Isotopically labeled PD-L1 D1 was expressed in modified Spizizen’s minimal media (59, 60), incorporating ^15^N-NH_4_Cl and ^13^C-glucose, and was refolded and purified as aforementioned.

### Expression and purification of anti-PD-L1 VHH

VHH coding regions were cloned into pET21a(+) with an N-terminal hexa-histidine tag and TEV cleavage site, using the In-Fusion HD Cloning Kit (Takara) following manufacturer’s instructions. VHHs were expressed as previously described (38) with some modifications. The pET21a VHH constructs were transformed into BL21(DE3) cells (New England Bioscience) and grown at 37 °C and protein expression induced by 1 mM IPTG. VHHs (6 and 9) that showed soluble expression were induced for 16 h at 20 °C, whereas insoluble VHH (1, 3, 4, 10, 11, 13 and 14–22) were expressed for 4 h at 37 °C. After harvesting, cells were lysed using a cell disruptor (Constant Systems). For insoluble VHHs, inclusion bodies were collected and washed twice with 50 mM Tris pH 7.8, 10 mM EDTA, 2 M urea, 0.1% Triton X-100 and 5 mM DTT, and finally with the same buffer excluding Triton and urea. Washed Inclusion bodies were resolubilized in 6 M guanidinium hydrochloride (GuHCl) at 0.5 mg/ml and dialyzed twice into 50 mM Tris pH 7.8, 1 M GuHCl, 0.2 mM oxidized GSH, and 1 mM reduced GSH and subsequently dialyzed into PBS (140 mM NaCl, 2.7 mM KCl, 6.5 nM Na₂HPO₄, and 1.5 mM KH₂PO₄, pH 7.4). VHH refolds or cell lysates for soluble VHH (6 and 9) were loaded onto a Ni-NTA Superflow column (Qiagen) in PBS and 20 mM imidazole and eluted over a 12 column volume gradient from 20 to 500 mM imidazole. VHHs were finally purified using a HiLoad Superdex 75 16/600 gel filtration column (Cytiva) equilibrated in PBS.

### Expression and purification of the extracellular region of CD80

A construct encoding CD80 (1–235), containing an N-terminal signal peptide and a C-terminal hexa-histidine tag, was synthesized and cloned into pcDNA4.1 by GenScript. The plasmid was then transfected into expiCHO high titer cells (Thermo Fisher) and grown for 10 days at 30 °C and 120 rpm in the presence of kifunensine. Supernatants containing the secreted extracellular region of CD80 were purified essentially as described for the VHHs. A final purified protein containing residues 35 to 235 of CD80 after signal peptide cleavage was obtained.

### Protein biotinylation

PD-L1 was biotinylated using the EZ-Link NHS-PEG4-Biotin, No-Weigh Format procedure (Thermo Fisher), following the manufacturer’s protocol. A 1:1 M ratio of biotin/PD-L1 was used to achieve a biotin incorporation ratio of one per molecule. Purified proteins containing an avitag were biotinylated using a BirA biotinylation kit (Avidity) as described by the manufacturer.

### Phage library enrichment against PD-L1

A naïve llama VHH phage library was kindly provided by UCB Biopharma. Enrichment of the phage against PD-L1 was performed as described previously (61), with some modifications. Panning was performed with Nunc Maxisorp ELISA plates (Invitrogen), with 1 μM biotinylated PD-L1 immobilized on the plate using neutravidin (Thermo Fisher) and streptavidin (Thermo Fisher), for the first and second rounds of panning, respectively. Following incubation, unbound phage were removed by 5 or 20 washes with PBS-T buffer (PBS, pH 7.4, and 0.05% (v/v) Tween-20) for round 1 and 2, respectively. Bound phage was then eluted with 100 mM HCl and the released samples neutralized with 1 M Tris–HCl, pH 8.0.

### Isolation of anti-PD-L1 VHHs

Individual colonies of phage from round 2 of the panning were subjected to monoclonal rescue, as previously described (61). An ELISA assay was used to assess binding to PD-L1 D1D2 and PD-L1 D1. Nunc Maxisorp ELISA plates were coated with streptavidin at 5 μg/ml overnight. Fifty microliters of 1 μg/ml of either biotinylated PD-L1 D1D2 or PD-L1 D1 was added to each plate and incubated for 30 min. The plate was blocked with 300 μl/well of M-PBS (PBS and 2% (w/v) milk powder) and incubated for 1 h. Monoclonal phage preps were mixed 1:1 (v/v) in M-PBS added to individual wells and incubated for 1 h. Anti-M13-HRP antibody (Thermo Fisher) diluted (1:10,000) in M-PBS was added to all wells and incubated for 1 h. Following each incubation step, plates were washed three times with 300 μl/well of PBS-T. Fifty microliters of enhanced K-Blue TMB substrate (Neogen) was added to each well and the reaction allowed to proceed for 10 min. The reaction was stopped by adding 50 μl of 0.25 g/l sodium fluoride and the absorbance at 650 nm was measured using a Microplate reader (Versamax).

### VHH sequence analysis

Individual VHH clones that showed binding to PD-L1 D1D2 were sequenced by Eurofins Genomics. The protein sequences obtained were analyzed using Mega7 (62). Multiple sequence alignments of the unique VHHs were performed using ClustalW, initially with a BLOSUM scoring matrix, and gap opening/extension penalties of 10 and 0.1 (63). The VHH sequences were grouped into families using the Neighbour-Joining method with a Poisson model featuring uniform rates and pairwise deletion (64, 65).

### Characterization of anti-PD-L1 affinity by BLI

All BLI experiments were performed on an Octet QKe (ForteBio) at 25 °C with a shaking speed of 1000 rpm. Proteins for analysis were prepared in sample buffer (PBS, pH 7.4, 0.01% (v/v) Tween-20 and 0.1% (w/v) bovine serum albumin). The sensorgrams were obtained with a 60 s baseline, 180 s loading, 60 s baseline, 300 s association, and 300 s dissociation. PD-L1-Fc fusion protein (Invitrogen) was diluted to 5 μg/ml in sample buffer and loaded onto Protein G biosensors (Sartorius). Individual purified VHH proteins were diluted in sample buffer to between 10 μM and 0.3 nM. Raw data were corrected with double referencing and analyzed using Prism9 (GraphPad). The dissociation constants (*K*_*D*_) for PD-L1-VHH binding were derived from steady-state equilibrium analysis of the sensorgrams using the maximum response observed during association of the VHHs binding to PD-L1 Fc. The binding curves were fitted in Prism to a one-site specific-binding model. Additional kinetic analysis was performed on the sensorgrams obtained for VHHs 4, 6, 9, and 15 to determine k_on_, k_off_, and *K*_*D*_ values, using the global analysis model for association kinetics within the Prism9 software.

### BLI characterization of PD-1-PD-L1 binding in the presence of VHHs

A series of BLI experiments were used to assess the inhibitory effects of individual VHHs with respect to PD-L1 binding to PD-1. Sensorgrams were acquired with a 60 s baseline, 100 s loading, 60 s baseline, 240 s association, and 240 s dissociation. Avitagged PD-1 was immobilized on streptavidin biosensors (Sartorius) at 5 μg/ml. Biosensors were incubated in wells containing either 3 μM PD-L1 D1D2 or 3 μM PD-L1 D1D2 with a high concentration of VHH (20 μM). The concentration of VHH was saturating (>10× *K*_*D*_) for the majority of the VHHs (10/16), with even the VHH with the weakest *K*_*D*_ expected to bind to 80% of the free PD-L1 present. The binding responses observed were compared for free PD-L1 D1D2 and the series of VHH PD-L1 complexes. The sensorgrams were corrected with double referencing and processed using octet supplied software.

### Characterization of VHH binding to PD-L2 by BLI

Equivalent BLI experiments were also used to assess VHH binding to PD-L2. Sensorgrams were obtained with a 60 s baseline, 180 s loading, 60 s baseline, 300 s association, and 300 s dissociation. PD-L2-Fc (Generon) was immobilized at 2 μg/ml on Protein G biosensors (Sartorius). PD-L2 was then incubated with VHHs at a concentration of 10 μM.

### Minimal shift mapping of VHH and CD80 binding sites

NMR spectra were acquired from 125 μM uniformly ^15^N/^13^C labeled PD-L1 D1 in a 25 mM KH_2_PO_4_, pH 7.4, 25 mM NaCl, and 10 μM EDTA buffer containing 5% (v/v) D_2_O for both free PD-L1 and PD-L1 in the presence of a 20% molar excess of individual VHHs. Similar NMR spectra for CD80 bound PD-L1 D1 were obtained from a sample containing a 60% molar excess of CD80. All NMR data were acquired on a Bruker Avance III 600 MHz spectrometer equipped with a TCI cryoprobe. Two-dimensional ^15^N/^1^H TROSY spectra were obtained at 25 °C in the presence and absence of VHHs or CD80, with typical acquisition times of 80 ms for ^1^H and 50 ms for ^15^N. Data were processed in Topspin (Bruker Biospin Ltd) and analyzed using the NMRFAM-Sparky software (66). Additional three-dimensional ^15^N/^13^C/^1^H HNCO spectra were acquired for PDL1 D1 in complex with VHH1, 2, 4, 6, 9, 13, 17, 18, 20, and 22. Triple resonance acquisition times were 80 ms for ^1^H, 25 ms for ^13^C, and 18 ms for ^15^N. All datasets were nonuniformly sampled to 25% and reconstructed using the iterative shrinking thresholding algorithm within NMRpipe (67). Spectra were analyzed using the Sparky software package (66). Backbone assignments for PD-L1 D1 have been previously published (39) and were consistent with the NMR spectra collected. Minimal shift analysis was performed by comparing bound and unbound PD-L1 D1 spectra, for both two-dimensional and three dimensional experiments, as described previously (40). The surface area affected by antibody binding was determined as the sum of the solvent accessible surface for each residue that experienced a major shift following binding of a VHH (>0.08), with solvent accessibility calculated using the deposited crystal structure of PD-L1 PDB: 5C3T (6).

### Crystallization of selected PD-L1 VHH complexes

For crystallography, TEV protease was used to remove the hexa-histidine tag from VHH1 and 6. PD-L1 D1D2 or PD-L1 D1 were mixed with a 20% molar excess of VHH1 or VHH6 and the complexes purified using a HiLoad 16/600 Superdex 75 gel filtration column (Cytiva) equilibrated in a 20 mM Tris–HCl, pH 7.5, and 50 mM NaCl buffer. All crystals were obtained by the vapor diffusion method at 19 °C by mixing equal volumes of protein plus well solution. VHH1⋅PD-L1 D1D2 crystals formed at 20 mg/ml in 0.1 M magnesium acetate, 0.1 M Mops, pH 7.5, and 12% (w/v) PEG 8000. VHH1⋅PD-L1 D1D2 crystals were cryoprotected in crystallization mother liquor and 25% ethylene glycol. VHH6⋅PD-L1 D1 crystals formed at 20 mg/ml and were optimized from a Morpheus (Molecular Dimensions) condition containing 0.1 M Hepes:Mops, pH 7.5, 0.1 M carboxylic acids, 10% (w/v) PEG 20000, and 20% (v/v) PEG 500-MME.

### Data collection and X-ray structure determination

X-ray diffraction data from VHH1⋅PD-L1 D1D2 and VHH6⋅PD-L1 D1 crystals were collected by the autonomous European Synchrotron Radiation Facility (ESRF, Grenoble) beamline MASSIF-1 (68), using automatic protocols for the location and optimal centering of crystals. All diffraction data were processed using XDS (69) and AIMLESS (70) from the CCP4 suite (71). Homology models of VHH1 and 6 were prepared using SWISSMODEL (72), using deposited VHH structures 5M3O (73) and 5IML (74), respectively. Structures of the VHH⋅PD-L1 complexes were determined by the molecular replacement program PHASER (75), using the VHH homology models and the published PD-L1 structure (PDB: 3BIK (42)) as search models. Atomic models were built using Coot (76) and refined using Refmac (77, 78). X-ray data collection and refinement statistics are given in Table 1. Structures were analyzed using PISA (79). Changes in solvent accessibility for both PD-L1 and VHHs were determined using PISA, which allowed comparison of the solvent accessibility of residues from the determined PD-L1/VHH crystal structures in the complexes and isolated proteins.

**Table 1:** X-ray data and refinement statistics

Data collection	VHH1⋅PDL1_D1D2	VHH6⋅PD-L1 D1
Wavelength (Å)	0.965459	0.965459
Space group	I 2_1_ 3	I 4_1_ 2 2
Cell dimensions		
a, b, c (Å)	149.512, 149.512, 149.512	99.575, 99.575, 171.515
α, β, γ (^o^)	90, 90, 90	90, 90, 90
Resolution (Å)	47.28–2.2	43.10–1.60
R_merge_	0.072 (1.930)	0.055 (1.119)
CC1/2	0.993 (0.287)	0.999 (0.536)
I/σI	10.2 (0.8)	15.5 (1.5)
Completeness (%)	99.9 (100)	99.9 (100)
Redundancy	7.6 (7.1)	5.6 (5.6)
Matthews coefficient	3.68	3.90
Solvent content (%)	66.16	68.47
Refinement		
No. of reflections	214,571 (17,319)	319,462 (15,678)
No. of unique reflections	28,212 (2424)	56,912 (2791)
R_factor_/R_free_ (%)	20.8/24.5	16.2/18.5
Wilson B-factors (Å)	62.95	21.14
B-factors (Å)		
Protein	75.27	25.12
Solvent	64.12	42.25
RMSDs		
Bond lengths (Å)	0.0135	0.0180
Bond angles (°)	1.98	2.138
Ramachandran plot		
Most favored (%)	95.45	96.65
Allowed (%)	3.96	3.35

Values shown in parenthesis correspond to the outer shell.

### CD80 AlphaFold model

The amino acid sequences for human PD-L1 (18–234) and CD80 (35–235) were submitted to AlphaFold-Multimer (48, 49) to produce a model of the complex. The top ranked AlphaFold model of CD80 bound to PD-L1 was compared to the solved crystal structure of PD-L1 bound to a high affinity variant of CD80 (PDB: 7TPS) (37) using DockQ (80) and the PyMOL Molecular Graphics System, Version 1.2r3pre, Schrödinger, LLC.

## Data availability

The VHH1⋅PD-L1 D1D2 and VHH6⋅PD-L1 D1 crystal structures reported here are available in RSCB PDB under accession codes 8AOM and 8AOK, respectively.

BioLayer Interferometry sensorgrams can be provided upon request.

## Supporting information

This article contains supporting information (6, 37).

## Conflict of interest

The authors declare that they have no conflicts of interest with the contents of this article.
