# Supplementary material for: Identification, binding, and structural characterization of single domain anti-PD-L1 antibodies inhibitory of immune regulatory proteins PD-1 and CD80
Source: J Biol Chem. 2022 Dec 5;299(1):102769. doi: 10.1016/j.jbc.2022.102769 (PMC9811221; doi:10.1016/j.jbc.2022.102769)
Supplement: Supplemental Table S1 [file mmc1.docx]

| **VHH** | **K_on_ (M^-1^s^-1^)** | **K_off_ (s^-1^)** | **K_D_ (nM)** | **K_D_ (nM)**  **steady state analysis** |
| --- | --- | --- | --- | --- |
| 4 | 5.9 ± 0.15 x 10^5^ | 2.9 ± 0.08 x 10^-2^ | 48 ± 0.85 | 59 ± 14 |
| 6 | 5.4 ± 0.19 x 10^5^ | 2.2 ± 0.11 x 10^-2^ | 40 ± 0.98 | 44 ± 11 |
| 9 | 3.0 ± 0.54 x 10^5^ | 4.1 ± 0.18 x 10^-3^ | 1.3 ± 0.05 | 0.7 ± 0.1 |
| 15 | 1.9 ± 0.34 x 10^5^ | 1.6 ± 0.04 x 10^-2^ | 8.9 ± 0.10 | 9.2 ± 1.2 |

Supplementary Table 1 A summary of the kinetic analysis carried out for selected high affinity VHHs binding to PD-L1 by BLI. The k_on_, k_off_ and K_D_ values were determined from the BLI sensorgrams using the global analysis model “association kinetics” within Prism version 9. Errors included are the standard error of the mean calculated for k_on_, k_off_ and K_D_. The K_D_ values determined for the selected VHHs binding to PD-L1 by steady state analysis of the sensorgrams are also including for comparison (Figure 3A).
