# Supplementary material for: Identification, binding, and structural characterization of single domain anti-PD-L1 antibodies inhibitory of immune regulatory proteins PD-1 and CD80
Source: J Biol Chem. 2022 Dec 5;299(1):102769. doi: 10.1016/j.jbc.2022.102769 (PMC9811221; doi:10.1016/j.jbc.2022.102769)
Supplement: Supplemental Figures S1–S3 [file mmc2.docx]

# Supporting Information


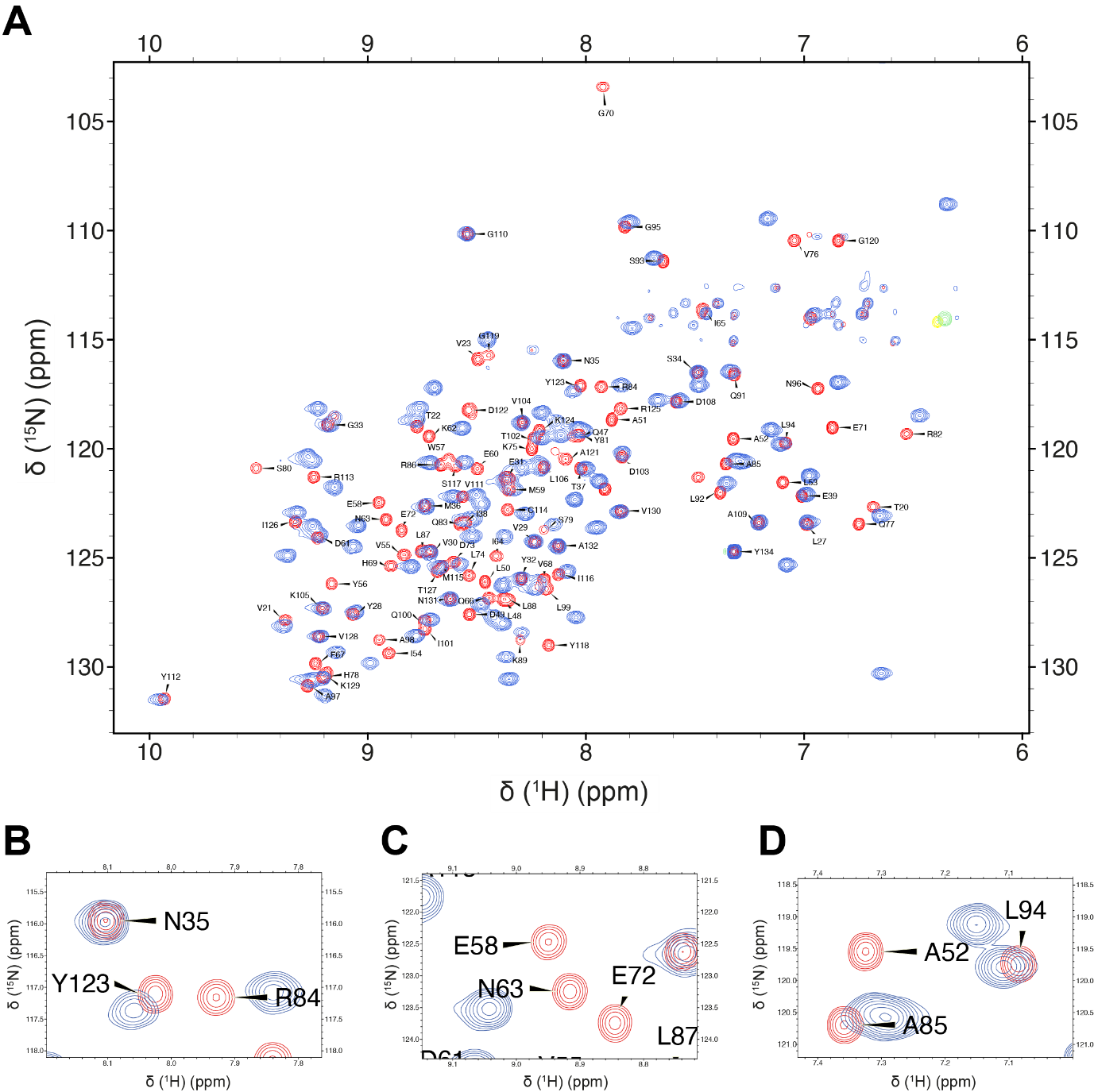


Supplementary Figure 1 ^15^N/^1^H TROSY-HSQC spectra of free and VHH10 bound PD-L1 D1. (A) The complete spectrum for free PD-L1 D1 is shown in red and in blue for VHH1 bound PD-L1 D1. Assignments of PD-L1 D1 are indicated for the free protein. (B-D) Expansions of selected regions of the spectral overlays highlighting significant shifts induced by VHH10 binding for residues such as Y123, E58 and A52.


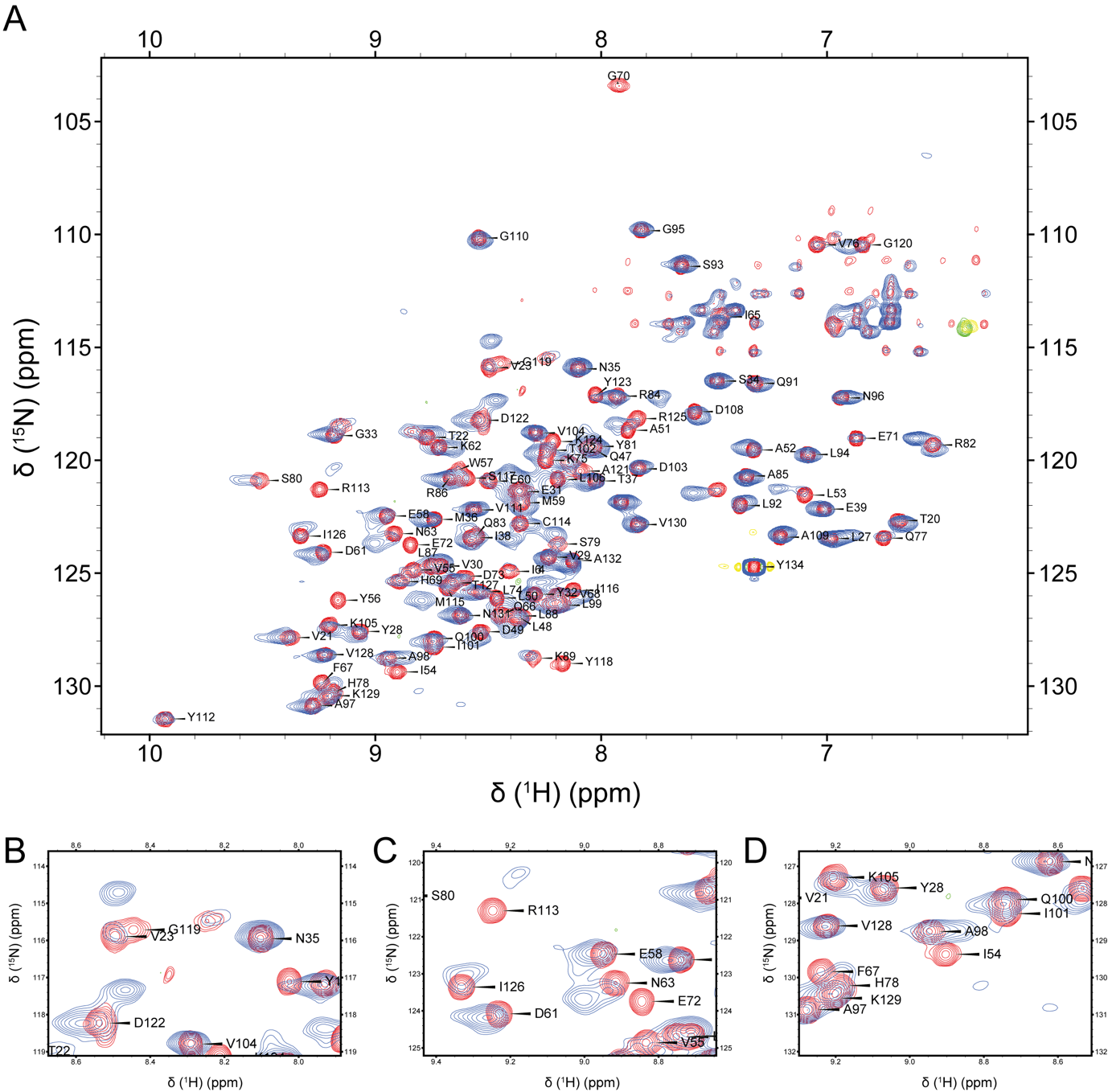


Supplementary Figure 2 ^15^N/^1^H TROSY-HSQC spectra of free and CD80 bound PD-L1 D1. (A) The complete spectrum for free PD-L1 D1 is shown in red and in blue for CD80 bound PD-L1 D1. Assignments of PD-L1 D1 are indicated for the free protein. (B-D) Expansions of selected regions of the spectral overlays highlighting significant shifts induced by CD80 binding for residues such as G119, R113 and I54.


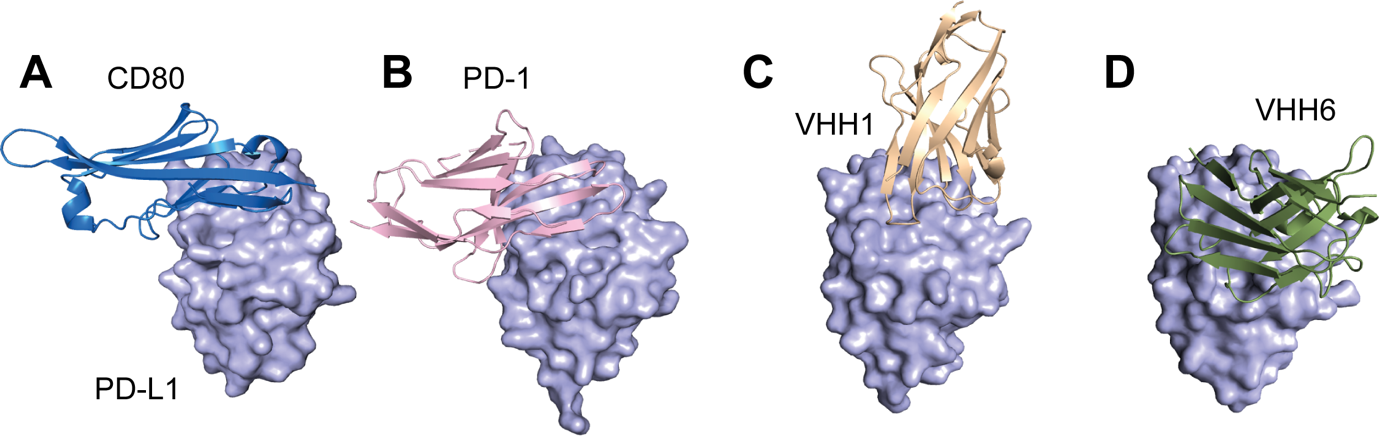


Supplementary Figure 3 Comparison of CD80, PD-1, VHH1 and VHH6 binding to PD-L1 D1. The surface view of PD-L1 is shown in light blue. PD-L1 is in the same orientation for all images. Ribbon representations of (A) CD80 IgV variant (dark blue) (PDB:7TPS (37)), (B) PD-1 (pink) (PDB:4ZQK (6), (C) VHH1 (light orange) and (D) VHH6 (green).
